# A Low-Temperature Micro Hotplate Gas Sensor Based on AlN Ceramic for Effective Detection of Low Concentration NO_2_

**DOI:** 10.3390/s19173719

**Published:** 2019-08-28

**Authors:** Wen-Jie Zhao, Dan Xu, Yin-Sheng Chen, Xuan Wang, Yun-Bo Shi

**Affiliations:** 1 Higher educational key laboratory for Measuring & Control Technology and Instrumentations of Heilongjiang Province, Harbin University of Science and Technology, Harbin 150080, China; 2 State Key Laboratory Breeding Base of Dielectrics Engineering, Harbin University of Science and Technology, Harbin 150080, China; 3 Key Laboratory of Quantum Manipulation & Control of Heilongjiang Province, Harbin University of Science and Technology, Harbin 150080, China

**Keywords:** gas sensor, ceramic micro hotplate, nitrogen dioxide, simulation analysis, low concentration

## Abstract

Air pollution is one of the major threats to human health. The monitoring of toxic NO_2_ gas in urban air emission pollution is becoming increasingly important. Thus, the development of an NO_2_ sensor with low power consumption, low cost, and high performance is urgent. In this paper, a planar structural micro hotplate gas sensor based on an AlN ceramic substrate with an annular Pt film heater was designed and prepared by micro-electro-mechanical system (MEMS) technology, in which Pt/Nb/In_2_O_3_ composite semiconductor oxide was used as the sensitive material with a molar ratio of In:Nb = 9:1. The annular thermal isolation groove was designed around the heater to reduce the power consumption and improve the thermal response rate. Furthermore, the finite element simulation analysis of the thermal isolation structure of the sensor was carried out by using ANSYS software. The results show that a low temperature of 94 °C, low power consumption of 150 mW, and low concentration detection of 1 to 10 ppm NO_2_ were simultaneously realized for the Nb-doped In_2_O_3_-based gas sensor. Our findings provide a promising strategy for the application of In_2_O_3_-based sensors in highly effective and low concentration NO_2_ detection.

## 1. Introduction

Nitrogen dioxide is not only a highly toxic and dangerous chemical gas but also an important petrochemical raw material and space propulsion fuel. It is also one of the main air pollution gases emitted by motor vehicle exhaust. It has an important impact on environmental pollution and human physical and mental health [1,2]. NO_2_ has strong oxidation and is easily transformed into unstable NO*x*, whose concentration is greatly affected by temperature and humidity. It is an important component of acid rain and becomes a difficult point in sensor detection [3]. Compared with traditional sensors, low power consumption, low cost, and portable application are the key factors for the wide application of NO_2_ gas sensors [4]. At present, the detection method of the NO_2_ gas sensor mainly adopts an electrochemical type and semiconductor type [5,6], while the NO_2_ gas detection method in an atmospheric environment is chemiluminescence and differential absorption spectroscopy, with the disadvantages of a high detection cost and complex operation, which cannot meet the requirements of real-time on-line detection, thus limiting its wide application [7,8]. In the case of traditional sensor monitoring, improving sensor manufacturing performance, signal detection level, and data processing model are the most promising ways to achieve low cost and fast, portable, and effective detection [9,10].

Sensors based on metal oxide semiconductors (MOS) have been widely investigated for NO_2_ detection, such as SnO_2_, TiO_2_, ZnO, WO_3_, and In_2_O_3_, etc. [11,12,13], which can effectively improve gas sensitivity characteristics through hybridization and modification with the advantages of a simple method, low cost, and wide detection range. Among them, SnO_2_-based gas sensors are the most commonly used for the detection of flammable gases. For example, SnO_2_-based CH_4_ sensors have attracted considerable attention due to their high sensitivity, high chemical stability, and low cost. However, the ideal working temperature of SnO_2_-based gas sensors is generally above 300 °C; it will undoubtedly bring high power consumption, which is unexpected in the application of sensors [14]. In the case of NO_2_ detection, a low working temperature is also required in order to achieve relatively low power consumption and improve the temperature reliability of the sensor. As an ideal gas sensing material, In_2_O_3_ has good development prospects for low temperature gas sensing detection of oxidizing gases, like NO_2_ [15,16]. To improve the NO_2_ gas sensing performance of In_2_O_3_-based materials, a lot of dopants and catalysts have been exploited and their role in NO_2_ sensing behavior has been investigated [17,18]. Among these dopants and catalysts, Nb is mainly employed to improve the conductivity and stability of metal oxides [17,19,20,21], and Pt is used to enhance the sensing response and selectivity of sensors [11,18,22].

In this paper, a micro hotplate NO_2_ sensor with an annular heating electrode based on AlN ceramic substrate was designed and fabricated by MEMS technology, in which Pt/Nb/In_2_O_3_ composites were used as the sensitive material and Nb-doped In_2_O_3_ with a molar ratio of In:Nb = 9:1 was prepared by chemical deposition. Finite element thermal simulation analysis of the thermal isolation structure of the sensor was carried out to verify the rationality of the thermal field distribution in the heating region. The thermal response characteristics of the micro hotplate sensor at different power consumptions were studied. The gas sensing properties of the sensor to low concentration and high concentration NO_2_ were investigated and analyzed in detail. The prepared micro hotplate sensor exhibits good NO_2_ sensing properties, with a low temperature, low power consumption, and low detection limit.

## 2. Thermal Structure Design and Simulation Analysis

### 2.1. Thermal Structure Design of Micro Hotplate Gas Sensor

The thermal structure design is the key for micro hotplate gas sensors. In order to achieve low power consumption, the “sandwich” structure, in which the metal heater is between the upper and lower insulation layers to reduce power consumption by reducing the area and volume of the heating region, is used in most of traditional silicon-based micro hotplate sensors. However, there are still problems in the thermal stress mismatch and process compatibility between the insulating layer with low thermal conductivity and silicon substrate with high thermal conductivity [23,24]. Fortunately, there are obvious advantages of using AlN over silicon with an insulating layer. As is well known to all, the ratio of the thermal expansion coefficient of Pt (9.0 × 10^−6^/K) to AlN (4.5 × 10^−6^/°C) is smaller (or closer to each other) than that of the silicon substrate (2.5 × 10^−6^/K) with an insulating layer. In this way, the thermal stress of the Pt film electrode is smaller and hence the adhesion between the Pt film and AlN ceramic substrate is better, which is beneficial to improving the quality of the film substrate. 

The micro hotplate sensor is designed by a plane structure; that is, the heater electrode and signal electrode are in the same plane, which not only simplifies the process but also solves the problem of thermal stress mismatch and improves the compatibility of sensitive materials. 

**Figure 1:**
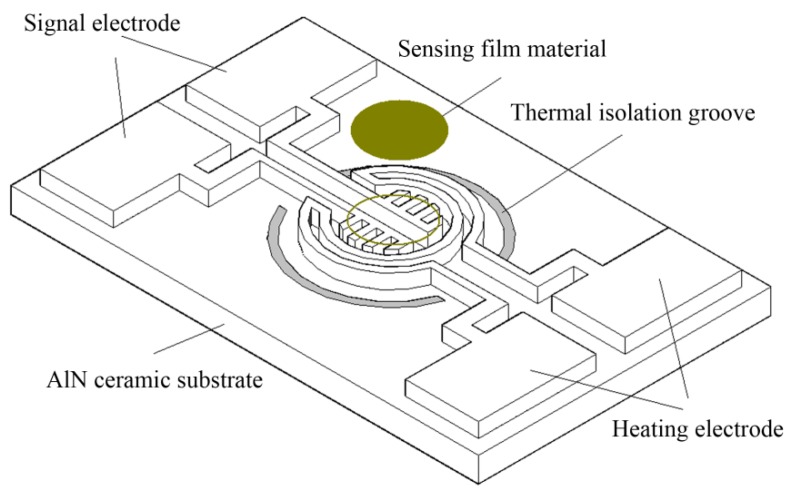
Structural schematic of the ceramic micro hotplate sensor.

Figure 1 shows a structural schematic of the ceramic micro hotplate sensor. As shown in Figure 1, the ceramic micro hotplate sensor uses AlN ceramic with good mechanical and thermal properties as the substrate, the Pt film graphic structure on the substrate is the heater electrode and the signal electrode, and the heater electrode adopts the annular structure. The signal electrode is in the center of the heater electrode, which has the outer comb-shaped structure. The comb-shaped signal electrode is coated with semiconductor sensitive film, and two semi-annular thermal isolation grooves are etched around the heater electrode. The size of the micro hotplate sensor is 3 mm × 1.8 mm, the width of the thermal isolation groove is 200 μm, the width of the heater electrode and signal electrode is 50 μm, and the thickness of the Pt film is about 500 nm.

**Figure 2:**
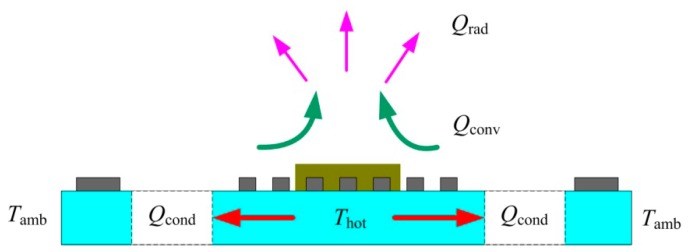
Schematic diagram of the thermal diffusion of ceramic micro hotplate sensor structure.

The thermal structure design of the micro hotplate sensor is the key factor affecting the thermal distribution of the temperature field. There are two main models for typical thermal field structure distribution, that is, rectangular and circular structure diffusion models. From the theory of thermal diffusion, it can be seen that the circular temperature field is more in line with the analysis of the theoretical model of thermal diffusion [25]. By optimizing the thermal field distribution by thermal structure design, the thermal efficiency and power consumption can be significantly improved, and the temperature gradient effect can be reduced. Figure 2 shows the schematic diagram of thermal diffusion of ceramic micro hotplate sensor structure. It can be observed from Figure 2 that there are three main thermal diffusion pathways in ceramic micro hotplate sensor structures, namely, heat conduction, heat convection, and heat radiation, in which heat loss caused by heat conduction is the main way.

The heat loss of the micro hotplate sensor is mainly based on the heat conduction of the membrane base, air thermal convection, and thermal radiation [26]. The thermal diffusion equation is as follows:

(1) $$Q_{tot}=Q_{cond}+Q_{conv}+Q_{rad},$$

where $Q_{cond}$ is the heat conduction loss, $Q_{conv}$ is the air thermal convection loss, and $Q_{rad}$ is the thermal radiation loss. Among the three heat dissipation pathways, the thermal isolation structure of micro hotplates belongs to a non-closed membrane structure, so the heat conduction of the membrane beam structure is the main heat dissipation way [27]. The equation is as follows:

(2) $$Q_{cond}=G_{m}\lambda_{m}(T_{hot}-T_{amb}),$$

where $G_{m}=NA_{beam}/l$ is the structural geometric factor, which is proportional to the cross-section, *A_beam_*, of the membrane beam and inversely proportional to the length, *l*, of the suspended beam. *N* is the number of membrane beams, *λ_m_* is the coefficient of heat conduction of the membrane base, and $T_{hot}$ and $T_{amb}$ are the heating region temperature and boundary temperature, respectively.

As revealed in Equation (2), $Q_{cond}$ can be reduced by decreasing the geometric factor, $G_{m}$; the substrate thermal conductivity, *λ_m_*; or the temperature difference, $T_{hot}-T_{amb}$. The reduction of $G_{m}$ can be realized by reducing the number of film beams, *N*, reducing the cross-section area, *A_beam_*, of membrane beams; or increasing the length, *l*, of film beams. Because the number of film beams, *N*, and the length of film beams, *l*, are limited by scale design, the most effective way is to reduce the thickness of the substrate and the cross-section area of thermal diffusion, *A_beam_*. Since the AlN ceramic substrate has high thermal conductivity and difficult to etch, in this paper, the laser etching thermal isolation groove is adopted to form a double-membrane beam suspension bridge structure, which can effectively reduce the diffusion cross-section area, *A_beam_*.

The heat loss in air is mainly air heat conduction and temperature difference heat flow exchange loss. The heat conduction coefficient of air at room temperature is $\lambda_{air}$ (25 °C) = 0.025 W·m^−1^·°C^−1^, and the natural convective heat transfer coefficient of air is $h_{f}$ = 10 W·m^−2^·K^−1^. In order to simplify the model, assuming that the heat source is a point, according to the spherical coordinate principle, the air heat conduction loss from the radius, $r_{i}$, to $r_{a}$ of the point heat source is shown in Equation (4) [28], and the heat flow exchange loss of air is shown in Equation (5):

(3) $$Q_{air}=Q_{cond}^{a}+Q_{conv},$$

(4) $$Q_{cond}^{a}=\frac{4\pi \lambda_{air}(T_{hot}-T_{amb})}{1/r_{i}-1/r_{a}},$$

(5) $$Q_{conv}=Ah_{f}(T_{hot}-T_{amb}).$$

In the thermal steady state, Equations (8) and (9) can be simplified into Equation (6) when boundary dimensions satisfy the condition of $r_{a}$ >> $r_{i}$:

(6) $$\begin{array}{c} Q_{air}=(4\pi r_{i}\lambda_{air}+Ah_{f})(T_{hot}-T_{amb})\\ =(G_{air}\lambda_{air}+G_{airf}h_{f})(T_{hot}-T_{amb}), \end{array}$$

where $\lambda_{air}$ is the heat conduction coefficient of gas, $h_{f}$ is the convective heat transfer coefficient of gas, $G_{air}$($G_{air}\approx 4\pi r_{i}$) and $G_{airf}$($G_{airf}\approx A$) are the structural geometric factors, in which *r_i_* is the effective radius of the heating area and *A* is the effective area of the heating region.

It can be seen from Equation (6) that the heat loss of air, $Q_{air}$, is proportional to the temperature difference, $T_{hot}-T_{amb}$. In fact, the air heat conduction coefficient, $\lambda_{air}$, is a variable dependent on the temperature gradient, $\Delta T=T_{hot}-T_{amb}$, and the thermal conductivity is related not only to the temperature in the heating region but also to the temperature distribution gradient. With the decrease of the effective radius, *r_i_*, of the heating region of the micro hotplate, the proportion of the heat flow exchange loss, $Q_{conv}$, decreases obviously, and therefore $Q_{cond}^{a}$ is the main one in air heat loss, $Q_{air}$.

According to the Stefan–Boltzmann law, thermal radiation loss is not only related to the geometric factors of the structure but also to the material and heating temperature, as shown in Equation (7). When the temperature is high, the thermal radiation loss cannot be ignored:

(7) $$Q_{rad}=G_{rad}\sigma \varepsilon (T_{hot}^{4}-T_{amb}^{4}),$$

where ε is the radiation coefficient, σ is the Boltzmann constant, and the structural geometric factors is $G_{rad}=A_{s}$ in which $A_{s}$ is the area of the heating region. Normally, when the temperature is not very high ($T_{hot}$ < 200 °C) and the heating area is small ($A_{s}$ < 0.5 mm^2^), the thermal radiation power consumption can usually be ignored below 1 mW.

### 2.2. Simulation Analysis of the Thermal Structure of a Micro Hotplate Sensor

**Figure 3:**
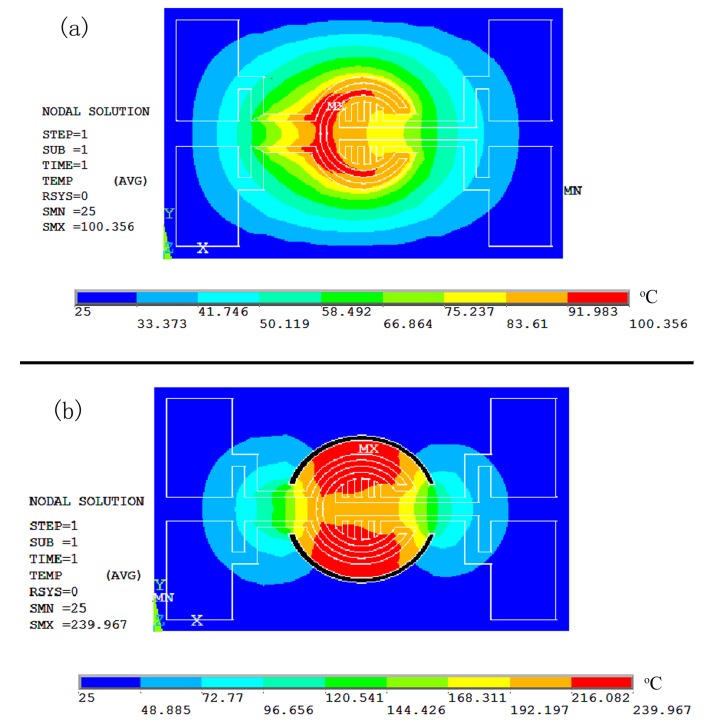
Temperature field distribution maps of finite element simulation of the thermal structure for the micro hotplate sensor (**a**) before thermal isolation and (**b**) after thermal isolation.

In order to verify the rationality of the thermal structure design of the micro hotplate, the finite element simulation of the thermal structure was carried out by using the ANSYS software. It was assumed that the ideal boundary condition was 25 °C, the chip scale of the sensor was 3 mm × 2 mm, AlN substrate thickness was 0.2 mm, the Pt film thickness was 500 nm, the thermal isolation through hole width was 50 μm, and the depth was 0.2 mm. The thermal conductivity of AlN and Pt film were 180 W·m^−1^·°C^−1^ and 73 W·m^−1^·°C^−1^, respectively. Under the condition of 1 × 10^7^ W/m^3^ load heat generation rate, Figure 3 shows temperature field distribution maps of the finite element simulation of the thermal structure for the micro hotplate sensor before thermal isolation (a) and (b) after thermal isolation. Here, “STEP“ represents the load step; “SUB“ represents the load substep;“TIME“ indicates the calibration time of the first load step, which is an interval and has no specific time meaning, only when it is related to speed or rate does it mean the real time; “RSYS“ is determines the Cartesian coordinate system; SMX and SMN are the abbreviations for Soltion Max and Soltion Min, respectively. Compared with the temperature field distribution of Figure 3a,b, it can be observed that the temperature efficiency is obviously improved by the design of the thermal isolation groove, and the maximum temperature (SMX) in the heating region increases from 100.36 °C before thermal isolation to 239.97 °C after thermal isolation, and the area of the effective temperature region increases obviously. 

**Figure 4:**
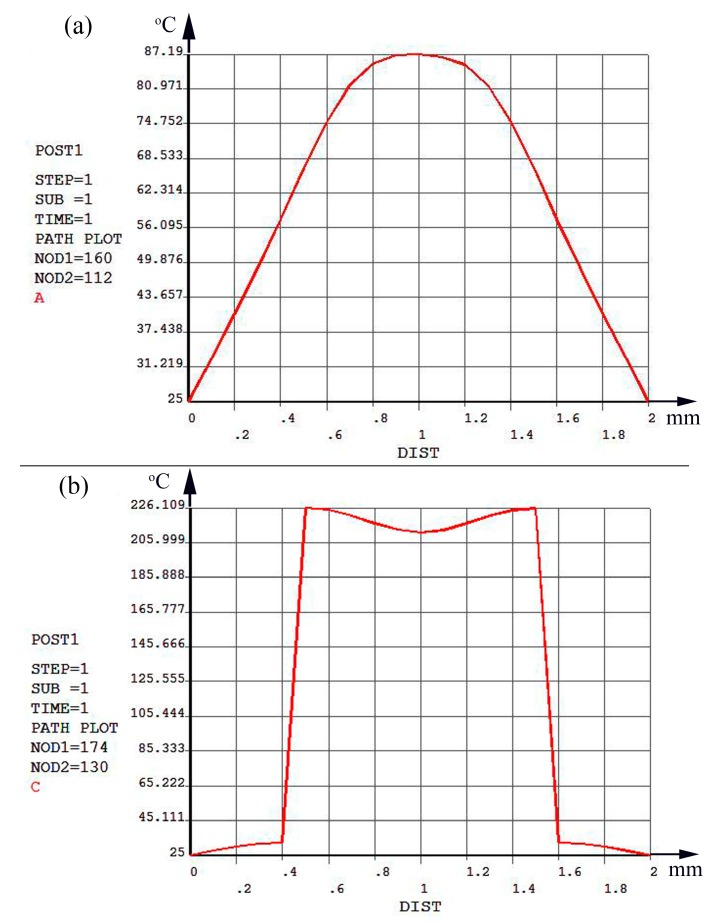
Radial temperature distribution curves (**a**) before thermal isolation (**b**) and after thermal isolation of the thermal structure simulation of a micro hotplate sensor.

Figure 4 is the radial temperature distribution curves before thermal isolation (a) and after thermal isolation (b) of the thermal structure simulation of the micro hotplate sensor, which is used to quantitatively analyze the temperature field distribution and temperature gradient in the center position of the sensor, where the profile is taken with respect to the pictures in Figure 3 top-down for both (a) and (b). As indicated in Figure 4a,b, the radial size of the effective equilibrium temperature region of the thermal isolation structure is about 1 mm, and the temperature gradient in the temperature region decreases from 15.10% before thermal isolation to 6.13% after thermal isolation. The thermal isolation design obviously reduces the temperature gradient effect, increases the area of the effective temperature region, improves the heat balance efficiency, and plays an important role in improving the temperature characteristics of gas sensing.

## 3. Experimental

### 3.1. Synthesis and Characterization of Sensitive Materials

The sensitive material was Pt/Nb_2_O_5_/In_2_O_3_, which is abbreviated as “Pt/Nb/In_2_O_3_”. Firstly, Nb-doped In_2_O_3_ oxide powder (Nb/In_2_O_3_) was prepared by chemical deposition. In(NO_3_)_3_·4.5H_2_O and Nb_2_O_5_ were dissolved in deionized water according to the molar ratio of In:Nb = 9:1, adding proper amounts of citric acid as a dispersing agent into the mixed solution, and then stirring at 50 °C for 2 h. Afterwards, ammonia was dripped into the solution to form a milky white precipitate. After separating the precipitate by low-speed centrifugation and washing it for 3 times, the white precipitate was dried at 120 °C for 2 h, and then was calcined at 500 °C for 2 h to obtain yellowish powder. Then, a certain amount of In/Nb oxide powder was used for agate grinding. A small amount of chloroplatinic acid (H_2_PtCl_6_) and terpineol were dripped into the Nb/In_2_O_3_ oxide powder to form the slurry. After that, the sensitive material slurry was coated onto the 0.25-mm radius circular area of the comb-shaped signal electrode outside the sensor. Finally, the Pt/Nb/In_2_O_3_ composite semiconductor oxide sensitive material was formed by sintering semiconductor slurry film at 500 °C for 2 h in a furnace. All chemicals used in the synthesis were analytic grade reagents without further purification.

**Figure 5:**
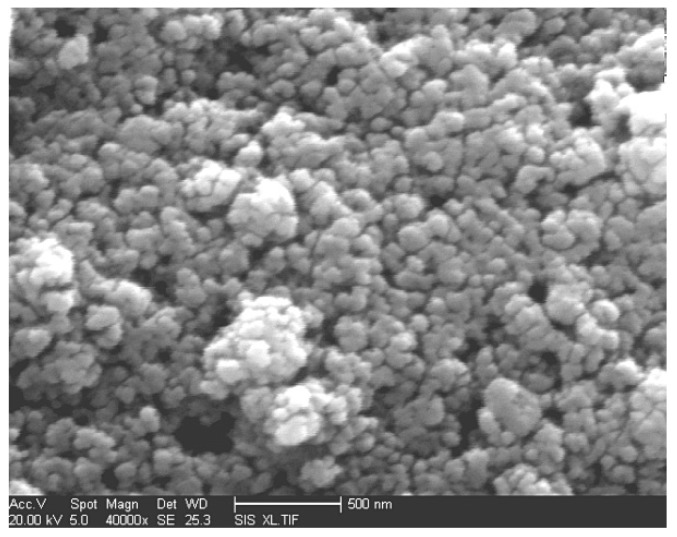
SEM images of the Nb/In_2_O_3_ sensing film.

Figure 5 is the SEM image of the Nb/In_2_O_3_ oxide sensing film. It was found that the composite oxide nanoparticles are regular, the agglomeration is less, and the interface of the particles is clear, which is very beneficial to the contact between the gas and the surface of nanoparticles.

### 3.2. Fabrication of the Gas Sensor

An AlN-based ceramic micro hotplate gas sensor chip was prepared by a flexible mechanical lithography peeling process and precision laser micro-machining etching. The electrode pattern of the Pt film sensor was formed on a 0.2-mm thick AlN ceramic substrate by a flexible mechanical lithography peeling process, in which the thickness of the Pt film was about 500 nm. The thermal isolation groove around the heater was prepared by laser etching. The width of the thermal isolation groove was about 50 μm, and its depth was 0.2 mm, which is the same as the thickness of the AlN substrate.

**Figure 6:**
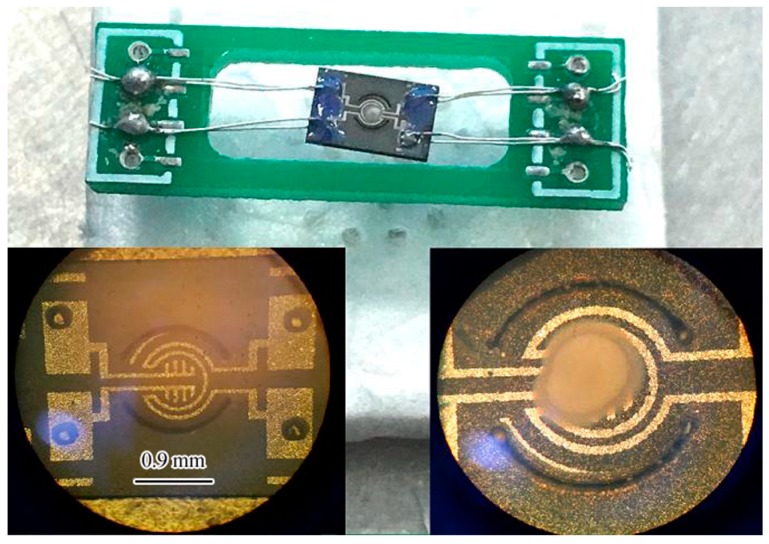
The real photograph of the sensor sealing structure.

Figure 6 is the real photograph of the sensor’s sealing structure. Both the heating electrode and the signal electrode used Pt film with good temperature characteristics and were distributed in the same plane. The comb-shaped signal electrode formed a circular sensitive area with a radius of 0.25 mm in the center of the annular heating electrode, and the composite semiconductor sensitive material Pt/Nb_2_O_5_/In_2_O_3_ was coated on the sensitive area in a thick film form. In order to reduce the contact thermal conductivity loss of the micro hotplate, the sensor chip adopted a suspension sealing structure, in which platinum wire with a diameter of 0.08 mm was used as the welding lead, and platinum slurry was used for sintering welding at 850 °C. In order to improve the welding quality of the chip and platinum wire, the chip pad was fixed by drilling and piercing, which significantly improved the welding reliability. Platinum wire lead welding adopted 850 °C platinum slurry sintering welding. This step was completed before the sensor sensitive material film formed, that is, the 500 °C sensitive material film sintering step began after 850 °C platinum slurry sintering welding. Consequently, the thickness of the sensitive material film (top gray-white region) after coating on the signal electrode was about 15 μm as shown in Figure S1 (see Supplementary Materials). 

## 4. Results and Discussion

### 4.1. Measurement of Thermal Response Characteristics

The length of the heating stability time of micro hotplate sensor is an important parameter to realize rapid detection. It is only when the thermal response reaches the thermal balance stability that the static gas sensing detection can be carried out, so the thermal response equilibrium rate is also an important factor affecting the gas sensitivity response rate. In addition, the thermal gradient effect is related to the thermal response rate. The thermal response rate of the substrate with high thermal conductivity is fast and the thermal gradient effect is small, but the heat conduction loss is large, and the substrate material with low thermal conductivity is just the opposite. In order to increase the thermal response rate and reduce the influence of the thermal gradient effect, the thermal isolation groove is designed around the heating region of the sensor substrate. In this way, the reduction can be realized in the heat conduction loss and decrease the thermal diffusion path at the same time, which will reduce the thermal gradient and increase the heat balance rate. In addition, to measure the temperature characteristics of the sensor, we designed a temperature sensor with the same structure as the prepared sensor, as shown in Figure S2 (see Supplementary Materials).

**Figure 7:**
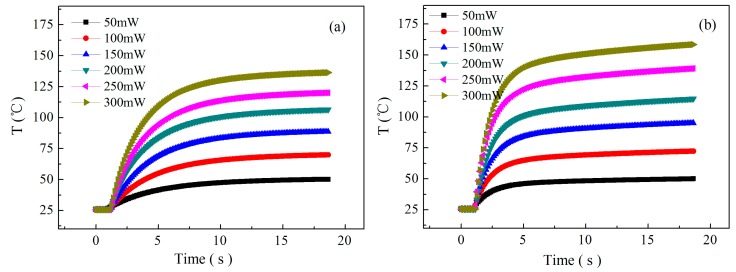
The thermal response characteristic curves of the sensor at different power consumptions (**a**) before thermal isolation and (**b**) after thermal isolation.

Figure 7 shows the thermal response characteristic curves of the sensor at different power consumptions before thermal isolation (a) and after thermal isolation (b). It can be seen that the larger the power consumption is, the longer the thermal response time will be, but after the thermal isolation design, the average thermal response time decreases from about 8 s before isolation to about 3 s after isolation, and the thermal response rate is obviously increased, and the thermal field temperature is also increased under the same heating power consumption, which indicates that the thermal isolation design has an important influence on increasing the thermal response rate and reducing the power consumption, and plays an obvious role in improving the thermal response characteristics.

As is well known to all, the response time of gas sensing is determined by the electron exchange rate between gas molecules and the surface of sensitive materials, which is affected by many factors, such as gas species, gas concentration, sensitive material properties, temperature, etc. Among them, the temperature level and temperature gradient of the sensor are the important factors that affect the gas sensing response rate, which determines the thermal stability time of the sensor heater and affects the gas sensing response time. As shown in Figure 7, the thermal response time of the sensor decreases from 8 to 3 s before and after thermal isolation, and the thermal response time of the sensor increased by 5 s, and hence the thermal performance of the sensor is obviously improved. However, the response time of the high concentration NO_2_ gas sensor is only about 40 s at 150 mW. Therefore, it still has great influence on the response time of high concentration NO_2_ gas sensors.

In addition, the temperature difference before and after thermal isolation shown in Figure 7 is indeed obviously smaller than the temperature difference in Figure 3. We believe that the reason may be discussed as follows: (1) The ideal parameters are set in Figure 3 of the simulation section and the simulation results do not take into account the actual boundary conditions and environmental effects; and (2) the actual measurement process needs encapsulation and connection, which will reduce the temperature effect. Although the design does not achieve the desired effect, it can be clearly seen from Figure 7 that thermal isolation can not only improve the thermal response rate but also improve the temperature effect, and play a significant role in reducing power consumption.

**Figure 8:**
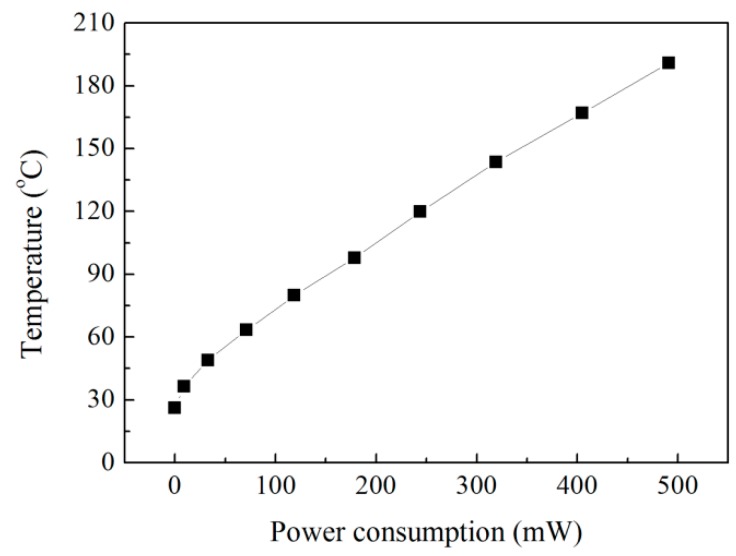
The relationship between the power consumption and temperature characteristics of the micro hotplate sensor.

There are two ways to reduce the power consumption of micro hotplate gas sensors. One is to develop low-temperature sensitive materials, the other is to miniaturize the structure size and optimize the structure design. In this paper, it is important to improve the efficiency of electrothermal coupling and accelerate the electron exchange rate of gas sensitive adsorption by optimizing the structure design to reduce the temperature gradient effect. Figure 8 shows the relationship between the power consumption and temperature characteristics of the micro hotplate sensor. Under the heating power consumption of 150 mW, the operating temperature of the sensor is only about 94 °C, and the operating temperature of the sensor is in the range of 90 to 200 °C at a power consumption range of 150 to 500 mW. There is an approximate linear relationship between power consumption and temperature.

### 4.2. Gas Sensing Response Characteristics of Low Concentration NO_2_

**Figure 9:**
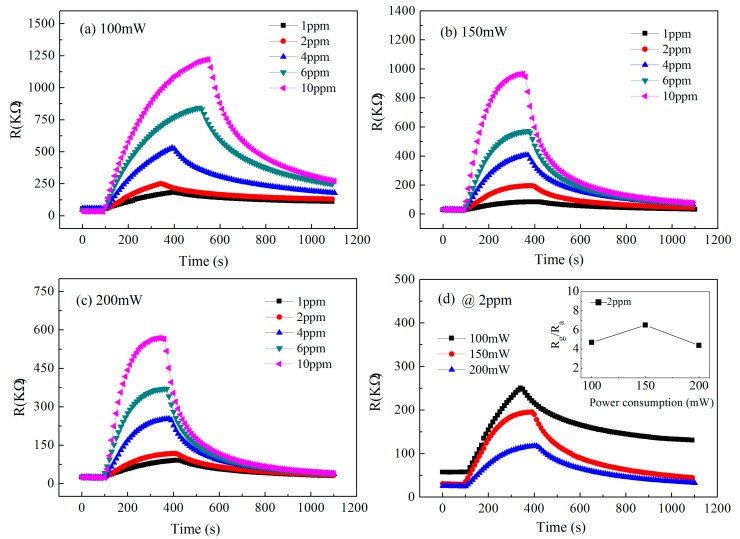
Gas sensing response characteristic curves of the micro hotplate sensor detecting low concentration NO_2_ at different power consumptions of (**a**) 100, (**b**) 150, and (**c**) 200 mW, and (**d**) at a fixed concentration of 2 ppm.

In static testing mode, the NO_2_ sensitivity response tests were carried out over a low concentration range of 1 to 10 ppm at different heating power consumption of 100, 150, and 200 mW, as shown in Figure 9a–c. It is found that the gas sensing response characteristics of low concentration NO_2_ at different heating power consumptions are obviously different, in which the recovery rate of the gas sensing response is relatively slow under the heating power consumption of 100 mW, so it is difficult to achieve the response balance, which indicates that the heating temperature of the micro hotplate sensor does not reach the ideal working conditions. However, the good gas sensing characteristics of low concentration NO_2_ are obtained at the power consumption of 150 and 200 mW. In this condition, the effective equilibrium can be achieved, and the response recovery rate is ideal. By comparing the 2 ppm NO_2_ sensing response curves of different power consumptions shown in Figure 9d, it is obvious that the response (*R*_g_/*R*_a_) of 2 ppm NO_2_ at 150 mW is the best, and the response and recovery time are about 180 and 300 s, respectively, so it has ideal comprehensive gas sensing characteristics.

Compared with Figure 9a–c, the gas sensing response characteristics of 1 to 10 ppm low concentration NO_2_ at different heating power consumptions are obviously different, in which the recovery rate of the gas sensing response is slow at 100 mW, and it is difficult to achieve the response balance, indicating that the heating temperature of the micro hotplate sensor below 100 mW does not reach ideal working conditions. However, it is easier to achieve good gas sensing characteristics for the micro hotplate sensor at 150 and 200 mW, the effective equilibrium state can be achieved, and the response recovery rate is ideal. As shown in the inset from Figure 9d, it can be seen that the response (*R*_g_/*R*_a_) at 150 mW to 2 ppm NO_2_ is the highest. Taking the time needed to reach 90% of the stable value and restoring the stable value of the gas sensing response as the response and recovery time, *T*_90_ and *t*_90_ are used to represent the response and recovery time, respectively. The response and recovery time of the micro hotplate sensor to 2 ppm NO_2_ at 150 mW are about *T*_90_ = 180 s and *t*_90_ = 10 min, respectively.

### 4.3. Gas Sensing Response Characteristics of High Concentration NO_2_

**Figure 10:**
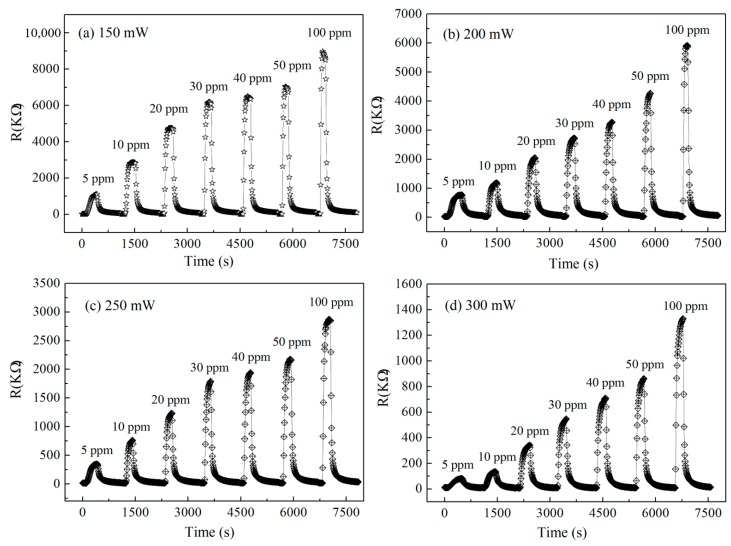
Gas sensing response and recovery property curves of the micro hotplate sensor detecting high concentration NO_2_ at different power consumptions of (**a**) 150; (**b**) 200; (**c**) 250; and (**d**) 300 mW in the range of 5 to 100 ppm.

**Figure 11:**
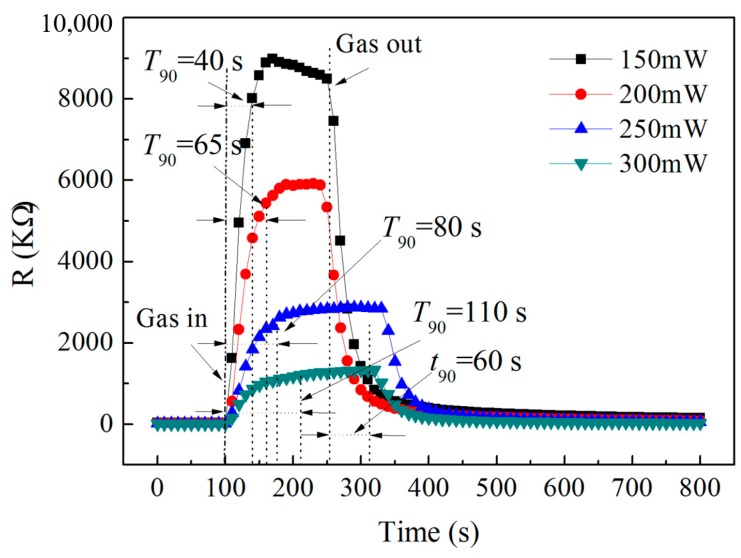
The gas sensing response characteristic curves at different heating power consumptions to 100 ppm NO_2._

Wide-range detection is one of the advantages of semiconductor micro hotplate gas sensors over other types of sensors. The above results of the gas sensing response of low concentration NO_2_ allow us to confirm the optimal power consumption of 150 mW. To further confirm that the good gas sensing characteristics can be achieved in a wider concentration range for In/Nb/Pt thick-film micro hotplate sensors, the NO_2_ sensing response and recovery property curves of the sensor were also carried out in the concentration range of 5 to 100 ppm at different heating power consumptions of 150, 200, 250, and 300 mW as shown in Figure 10. With the increase of the heating power consumption, the working temperature will increase accordingly, the resistance of the sensor in air decreases obviously, and the gas sensitivity response rate increases obviously with the increase of the gas concentration measured. In order to further illustrate the effect of power consumption on the gas sensing response time, Figure 11 shows the gas sensing response characteristic curves at different heating power consumptions to 100 ppm NO_2_, in which the response time, *T*_90_, is about 40 s at 150 mW and the recovery rate, *t*_90_, is about 60 s, and the response time is significantly slower with the increase of the heating power consumption.

As indicated in Figure 11, with the increase of the heating power consumption, the response of the sensor to 100 ppm NO_2_ shows a downward trend, in which the sensitivity curve of 200 and 250 mW is intersecting, and the sensitivity is higher at 150 mW, which is consistent with the response change of 2 ppm NO_2_ in Figure 9d, indicating that the sensor has higher sensitivity at 150 mW.

**Figure 12:**
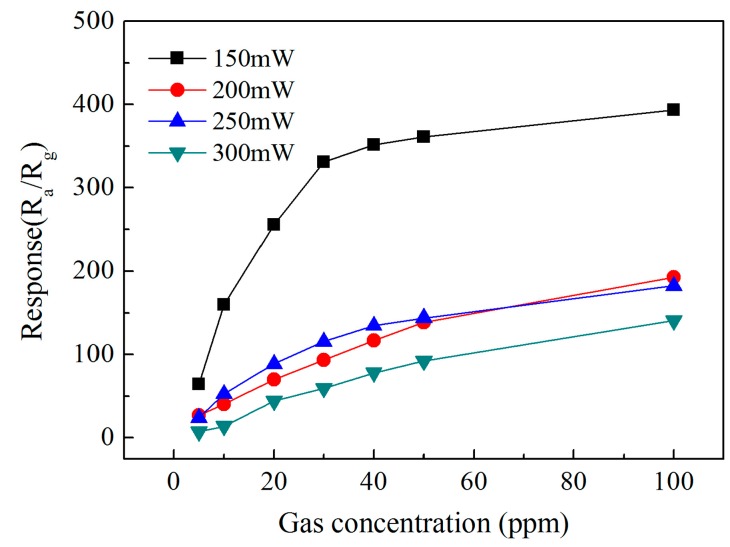
Variation of the response to NO_2_ with the gas concentration at different power consumptions.

In order to further analyze the influence of different heating power consumptions on the gas sensitivity response characteristics, Figure 12 shows the sensitivity curve of the sensor of different heating power consumptions. As can be seen from Figure 12, with the increasing power consumption, the sensitivity of NO_2_ shows a downward trend, and the sensitivity is the highest at 150 mW, which indicates that the sensor has higher sensitivity and a faster response rate at 150 mW compared with other power consumptions.

Taking the abovementioned results into consideration, the attained Pt/Nb/In_2_O_3_ sensor has a low operating temperature of 94 °C and rapid response time of 180 s to 1 ppm NO_2_ at 150 mw power consumption, which is superior to several other gas sensors reported as In_2_O_3_-based sensors as listed in Table 1. Although it is slightly inferior compared with Fe-In_2_O_3_ and Pt-In_2_O_3_ MCs sensors, which exhibit a high response of 71 and 44.9 at a low NO_2_ concentration of 1 ppm, the response (2.87 to 1 ppm NO_2_) of the Pt/Nb/In_2_O_3_ sensor in our work is comparable to that of In_2_O_3_ nanowires (2.57 to 1 ppm NO_2_). Our findings provide a potential application for In_2_O_3_-based gas sensors in highly effective and low concentration NO_2_ detection.

## 5. Conclusions

In summary, a planar structural micro hotplate NO_2_ gas sensor based on AlN ceramic substrate with an annular heater was designed and prepared by MEMS technology, in which Pt/Nb/In_2_O_3_ composites were used as the sensitive material. Finite element simulation analysis of the thermal isolation structure of the sensor was carried out by using ANSYS software, which verified the rationality of the thermal isolation structure design and the feasibility of the preparation process. The thermal response characteristic tests of different power consumptions were investigated and analyzed, and the gas sensing response performance of the micro hotplate gas sensor was studied and discussed in detail over a low concentration range of 1 to 10 ppm and a high concentration range of 5 to 100 ppm under different heating power consumption conditions. The results demonstrate that the optimal operating temperature is 94 °C and the optimal heating power consumption is 150 mW. The low temperature, low power consumption, and low concentration detection of the prepared NO_2_ gas sensor provide a potential way for detecting air pollution.

## Figures and Tables

**Table 1:** Comparison of the sensing performance of various NO_2_ gas sensors based on In_2_O_3_.

Materials	Operation Temperature (°C)	Concentration (ppm)	Response (*R*_g_/*R*_a_)	Response Time (s)	Recovery Time (min)	Reference
In_2_O_3_ nanowires	250	1	2.57	N/A	N/A	[29]
Pt-In_2_O_3_	250	5	1904	330	14	[30]
Fe-In_2_O_3_	150	1	71	276	2.5	[31]
Pt/In_2_O_3_ MCs	40	1	44.9	N/A	7	[12]
Pt/Nb/In_2_O_3_	94	1	2.87	180	10	This work

## Supplementary Materials

The following are available online at https://www.mdpi.com/1424-8220/19/17/3719/s1, Figure S1: SEM sectional image of sensitive materials, Figure S2: The structure of temperature sensor.
