# Supplementary material for: A Low-Temperature Micro Hotplate Gas Sensor Based on AlN Ceramic for Effective Detection of Low Concentration NO2
Source: Sensors (Basel). 2019 Aug 28;19(17):3719. doi: 10.3390/s19173719 (PMC6749266; doi:10.3390/s19173719)
Supplement: Supplementary file 1 [file sensors-19-03719-s001.pdf]

A low Temperature Micro Hotplate Gas Sensor Based on AlN Ceramic for Effective Detection of Low Concentration NO_2

Wen-Jie Zhao [1, 2], Dan Xu [3, *], Yin-Sheng Chen [1], Xuan Wang [2] and Yun-Bo Shi [1,2]

The thickness of the sensitive material (top gray-white region) after coating on the signal electrode is about 15 μm as shown in Figure S1.

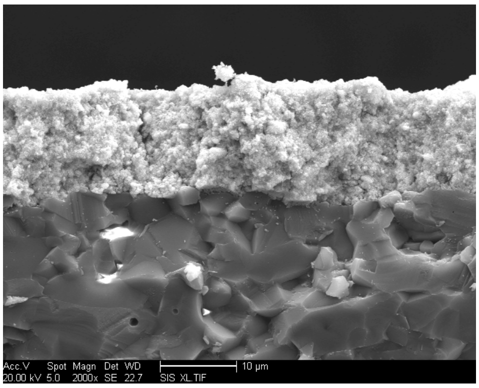

Figure S1 SEM sectional image of sensitive materials.

In order to measure the temperature characteristic of the sensor, we designed a temperature sensor with the same structure as the one of the sensor, as shown in figure S2, where the signal electrode in the sensor structure was replaced by a temperature electrode. The temperature characteristics of the sensor were measured by good temperature characteristics of the Pt film, and the specific temperature calibration details are not described in detail herein.

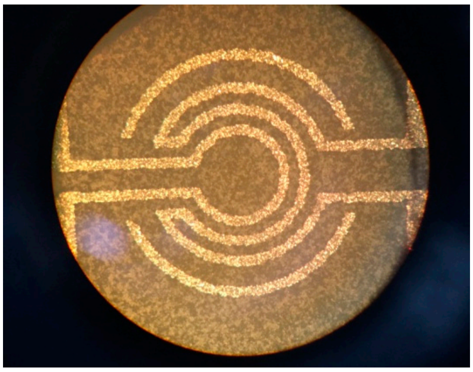

Figure S2 The structure of temperature sensor
